# WiFi Related Radiofrequency Electromagnetic Fields Promote Transposable Element Dysregulation and Genomic Instability in *Drosophila melanogaster*

**DOI:** 10.3390/cells11244036

**Published:** 2022-12-13

**Authors:** Ugo Cappucci, Assunta Maria Casale, Mirena Proietti, Fiorenzo Marinelli, Livio Giuliani, Lucia Piacentini

**Affiliations:** 1 Department of Biology and Biotechnology “C. Darwin”, Sapienza University of Rome, 00185 Rome, Italy; 2 National Research Council of Italy (CNR), Istituto di Genetica Molecolare (IGM), 40136 Bologna, Italy; 3 ICEMS-CIRPS (Centro Interuniversitario di Ricerca Per lo Sviluppo Sostenibile), 00038 Valmontone (Rome), Italy; 4 ECERI (European Cancer Environment Research Institute), 1000 Bruxelles, Belgium

**Keywords:** radiofrequency electromagnetic fields, transposable elements, *Drosophila melanogaster*

## Abstract

Exposure to artificial radio frequency electromagnetic fields (RF-EMFs) has greatly increased in recent years, thus promoting a growing scientific and social interest in deepening the biological impact of EMFs on living organisms. The current legislation governing the exposure to RF-EMFs is based exclusively on their thermal effects, without considering the possible non-thermal adverse health effects from long term exposure to EMFs. In this study we investigated the biological non-thermal effects of low-level indoor exposure to RF-EMFs produced by WiFi wireless technologies, using *Drosophila melanogaster* as the model system. Flies were exposed to 2.4 GHz radiofrequency in a Transverse Electromagnetic (TEM) cell device to ensure homogenous controlled fields. Signals were continuously monitored during the experiments and regulated at non thermal levels. The results of this study demonstrate that WiFi electromagnetic radiation causes extensive heterochromatin decondensation and thus a general loss of transposable elements epigenetic silencing in both germinal and neural tissues. Moreover, our findings provide evidence that WiFi related radiofrequency electromagnetic fields can induce reactive oxygen species (ROS) accumulation, genomic instability, and behavioural abnormalities. Finally, we demonstrate that WiFi radiation can synergize with Ras^V12^ to drive tumor progression and invasion. All together, these data indicate that radiofrequency radiation emitted from WiFi devices could exert genotoxic effects in *Drosophila* and set the stage to further explore the biological effects of WiFi electromagnetic radiation on living organisms.

## 1. Introduction

Transposable elements (TEs) are highly conserved mobile DNA elements that can move by themselves within a host genome and change their location both within the same chromosome and from a chromosome to another. Transposable elements occupy a large fraction of all eukaryotic genomes [1,2], and can be distinguished into two main classes based on their transposition mechanism [3]: class I TEs, also known as retrotransposons (RTEs), mobilize through a “copy-and-paste” mechanism using an RNA intermediate, and Class II TEs, which move via a conservative “cut-and-paste” transposition mechanism. Due to their mutagenic effects, TEs were initially considered as “junk DNA” or “selfish DNA” that potentially threatens genome integrity and stability [4]. In recent years, however, their function as inducers of genetic variability and motors of evolution was re-evaluated [5,6,7,8,9]. It is becoming clear, in fact, that TEs can have a huge impact on the structure and functions of most eukaryotic genomes [10,11,12,13]; moreover, since they are able to finely modulate and reprogram the expression of complex gene networks, transposable elements are excellent tools that can be used by genomes to functionally respond to environmental changes [14,15,16]. In the early 1980s, Barbara McClintock suggested for the first time that transposable elements could actively reprogram the gene regulatory networks of the host genome by finely modulating their response to specific environmental stimuli [17]; later, the activation of transposable elements in response to different types of stress such as temperature, exposure to UV rays, radiation, pathogen infection, and polyploidization was identified in *Drosophila* and other organisms [6,7,18]. Data recently produced in our laboratory have confirmed that emotional stress can also trigger the activity of TEs in the rodent hippocampus, suggesting that TEs may have a potentially decisive role in the development of post-traumatic stress disorders [19]. Stress-induced expression of TEs can improve the genome’s ability to flexibly cope with environmental changes and stress through two different mechanisms. First, the mutagenic activity of TEs could directly trigger genetic variability resulting in mutations, chromosomal rearrangements, and new functional regulatory elements [20]. Secondly, TE transcripts could greatly influence gene expression by producing small regulatory RNAs capable of modulating the expression profiles of non-adjacent genes in-trans [20,21]. However, for transposable elements to co-evolve with host genomes, their mobility must be finely regulated, as an uncontrolled transposition activity can cause genome instability and an altered expression of many genes with consequent deleterious effects on genome functions. Aberrant TE activation has been reported in both neurodevelopmental and neurodegenerative disorders [22,23]. Rett syndrome, for example, was the first genetic neurological disorder to be associated with a significantly increased long interspersed nuclear elements 1 (L1) somatic retro-transposition [24]. The uncontrolled activation of transposable elements has also been reported in ataxia-telangiectasia [25,26], macular degeneration [27], prion diseases [28], amyotrophic lateral sclerosis (ALS) [29,30], Aicardi–Goutières syndrome (AGS) [31], Alzheimer’s disease [32,33,34] and Huntington’s disease [35]. Bundo et al. also demonstrated that L1 copy number is increased in the prefrontal cortex cells and in pluripotent stem cells of schizophrenic patients, suggesting that upregulation of L1 activity could also contribute to the pathogenesis of schizophrenia [36].

Based on all these data correlating the activity of transposable elements to neurodegeneration, and since environmental stress can cause an uncontrolled and aberrant activation of TEs, the main purpose of this study was to investigate whether high frequency WiFi electromagnetic fields can trigger the activity of transposable elements. *Drosophila* was used as an experimental model. 

Electromagnetic fields of all frequencies are, in fact, one of the most common causes of rapidly increasing environmental pollution. All populations are exposed—to different extents—to electromagnetic fields, the intensity of which will continuously increase with the development of current technologies. In recent years, there has been considerable interest and controversial debate concerning the biological and health effects associated with the use of modern wireless devices that emit high frequency electromagnetic waves. The current western exposure limits for high frequency electromagnetic fields were established simply to protect biological tissues from “thermal effects” associated with exposure to RF radiation (ICNIRP, 1998; IRPA, 1988). These standards, however, do not consider the non-thermal biological effects associated with chronic RF exposure. Several studies provided experimental evidence that long term exposure to current environmental frequencies of wireless networks (2.4 GHz) may be associated with oxidative stress [37,38,39,40,41,42,43,44,45,46], male infertility [37,38,47,48,49,50,51,52,53,54], neuropsychiatric and behavioural disorders [42,45,47,48], direct damage to neuronal cells [55,56], DNA damage [37,49,52], fetal damage and impaired neuronal development [57], increased risk of neurodegenerative diseases [56], impaired microRNA expression in the brain [48], metabolic and endocrine system disorders [44,45,58]. The results of all these studies (reviewed in [59]), however, are difficult to interpret as most of them failed to explain the molecular mechanisms underlying these effects. 

Using *Drosophila* as an experimental model, we demonstrated that exposure to radiofrequency (RF) electromagnetic fields produced by WiFi technologies cause genome instability in neuronal tissues both through heterochromatin loss and transposable element dysregulation. Noteworthy, in this study we also showed that chronic exposure to WiFi radiation promotes tumor growth and metastatic behavior of non-invasive eye disc clones expressing the activated oncogene *Ras^V12^*.

## 2. Materials and Methods

### 2.1. Drosophila Strains 

The *Drosophila* stock used in this study were obtained from Bloomington *Drosophila* Stock Center (Indiana University, Bloomington, IN, USA): y^1^, w*; Dp(3;Y)BL2, P{HS-lacZ.scs}65E (#57371); *In(1)w^m4^* (#807). Fluorescently labelled tumor clones were produced in the eye discs as previously described [60] using the following strains: *ey-FLP1; act > y^+^ > Gal4, UAS-GFP; FRT82B, tub-Gal80* and *FRT82B, UAS-Ras^V12^*. The Ore-R stock have been kept in our laboratory for many years. All flies were raised at 25 °C on a standard cornmeal-sucrose-yeast-agar medium.

### 2.2. RF/EMF Exposure

Flies were exposed to the WiFi electromagnetic radiation inside a Transverse Electromagnetic Cell (TEM-CELL) (U.S. patent n. US5436603A) that enables the exposure of the samples to frequencies greater than 800 MHz with a characteristic impedance of 50 Ohm [61]. The TEM-CELL was a specially constructed copper box with the following dimensional characteristics: thickness 2.8 mm, total length 50 cm, sizes of the central body H 20 cm × L 25 cm × P 20 cm, and length of each pyramid 19 cm. The cell geometry and field propagation were previously described [62,63]. The WiFi 2437 MHz electromagnetic field was generated by a commercial router producing a frequency ranging between 2.39 and 2.49 GHz. The output of the router was modified excluding the propagating antennas and addressing the output signal, through a coaxial cable, directly to the TEM-CELL N connector. The TEM-CELL contains a “strip line”, a flat copper septum which divides the inner space into two parts and allows the electric field to propagate quite uniformly towards the sides of the box. The N coaxial connectors, at the ends of the septum, link the strip line to the router output. The TEM-CELL is terminated into a 50 Ohm load by the N connector. The impedance adapter minimizes the steady-state waves. When adjusted exactly, the system measured 85 dBm of return loss at 2437 MHz frequency. These were the necessary conditions favoring the best uniformity of the electric field with no differences higher than 2 dB in the TEM CELL inner space. A FSH-3 handheld spectrum analyzer with a tracking generator by Rohde/Schwarz (R&S s.n. 100929) was used to continuously detect the administered field. A 2437 megacycles-per-second field was applied continuously from the router to the TEM-CELL at −14.47 dBm. Perpendicular to the septum plane, the electric field was 1.35 V/m and 3.357 mA/m at a power density of 0.0048342 W/m^2^. The SAR calculation rate was 0.0608 W/Kg. This rate corresponds to an equivalent global exposure to the electric field of a standard man (height 175 cm, 70 Kg) of 53.55 V/m, lower than the exposure limit stated in the US FCC OET Bulletin n. 65/1997 or the reference level in the EU recommendation 1999/519/CE (61 V/m) for such frequencies, 61.4 and 61 V/m, respectively. The control samples were kept in a second TEM-CELL with the same dimension and characteristics of the first one, but were not connected to the router WiFi. Control flies were exposed to 0.10 V/m due to the electromagnetic background that induced a SAR calculation rate of 0.000334 W/Kg, corresponding to an exposure to the electric field for a standard man of 3.97 V/m. 

To evaluate whether the exposure caused any thermal increment in the chamber, a continuous temperature monitoring was performed in the cell and inside the chamber during the entire exposure time. To monitor the temperature, isolated thermocouples and a conventional alcohol thermometer were used. Thermo-robes were positioned inside the chamber in the upper plate of the TEM cell. During the experiments we detected no more than 0.1 °C degrees of temperature difference, so that the observed EMF effects were independent from thermal phenomena.

### 2.3. Heat Shock Treatment

Ten days-old adult wild-type Oregon-R flies were treated at 37 °C for 2 h and allowed to recover for 20 min at room temperature before collecting tissue samples.

### 2.4. Reverse Transcription and Quantitative RT-PCR (qRT-PCR)

Total RNA was purified according to the protocol supplied with Qiazol reagent (Qiagen, Hilden, Germany) and, after DNAse treatment (RNase-Free DNase Set (Qiagen, Hilden, Germany), RNA was extracted with Ambion^®^ Acid Phenol:Chloroform:IAA (AM9732). Total RNA was reverse transcribed using oligo dT and SuperScript Reverse Transcriptase III (Invitrogen) according to the manufacturer’s protocol. The qPCR reactions were carried out with QuantiFast SYBR Green PCR Kit (Qiagen, Hilden, Germany) according to the manufacturer’s protocol. For the quantification of transcripts, we used the 2^−ΔΔCt^ method [64] by comparing the amount of transcript to the rp49 transcript which exhibited a stable expression pattern across all tissues and experimental conditions tested in this study. The BestKeeper software (https://www.gene-quantification.de/bestkeeper.html, last accessed on 3 June 2022) was used to calculate rp49 expression stability with respect to other reference genes. qRT-PCR experiments were performed at least in two independent biological replicates each with three technical replicates. The primers used were:
blood FTGCCACAGTACCTGATTTCGblood RGATTCGCCTTTTACGTTTGCcopia FTGGAGGTTGTGCCTCCACTTcopia RCAATACCACGCTTAGTGGCATAAAgypsy FCTTCACGTTCTGCGAGCGGTCTgypsy RCGCTGCAAGGTTACCAGGTAGGTTCHet-A FACTGCTGAAGCTCGGATTCCHet-A RTGTAGCCGGATTCGTCATATTTChobo FAAACTGTTCTGGACGGATGGhobo RTTATGGCGGGATAAATTGGAI-element FCAATCACAACAACAAAATCCI-element RGGTGTTGGTGTGGTTGGTTGR2 FATGCTCCCGAAACAACAAACR2 RGCACTGCAGACTTGGTTCAA1360 FTCGTGCAAGACAATGAGAGG1360 RGCAACTGGATCCCTTAGCAAGstD1 FCGCGCCATCCAGGTGTATTTGstD1 RCTGGTACAGCGTTCCCATGTcatalase FCAACCCCTTCGATGTCACCAcatalase RTCTGCTCCACCTCAGCAAAGsod1 FGAACAGGAGAGCAGCGGTAsod1 RTGCCATACGGATTGAAGTGCsod2 FCAAACTGCAAGCCTGGCGsod2 RCTGGTGGTGCTTCTGGTGAThsp83 FCAGCTGGTCTCTGTCACCAAhsp83 RCTCTCGAACTTGGCCTTGTChsp70 FCAACCTATCCATCAACCCAGAChsp70 RACGTAGCTCTCCAGAGCATTTChsr-ω FTCTGCGACCGTGACTGAGATChsr-ω RCAATCCGCACAATCAATCTGArp49 FGCGCACCAAGCACTTCATCrp49 RTTGGGCTTGCGCCATT

Statistical significance was determined by the Unpaired *t*-test using GraphPad Prism version 6.00 (La Jolla, CA, USA). A *p* value ≤ 0.05 was considered statistically significant.

### 2.5. Western Blotting

Protein extracts were fractionated by 12% SDS-PAGE and electroblotted onto immobilon-P polyvinyl difluoride membranes (Bio-Rad Laboratories S.r.l., Milan, Italy) in CAPS-based transfer buffer (10 mM CAPS pH 11, 10% methanol) in a semi-dry transfer apparatus (Amersham Biosciences, Amersham, UK). The membranes were blocked with 5% nonfat dry milk in tris-buffered saline with Tween 20 (TBST) buffer (20 mM Tris pH 7.5, 150 mM NaCl, 0.1% Tween 20) and incubated with the following antibodies diluted in TBST: mouse anti-H3K9me2 (1:1000, Active Motif, Carlsbad, CA, USA #39683), mouse anti-H3K9me3 (1:1000, Active Motif, Carlsbad, CA, USA #61013), mouse anti-H3K27me3 (1:1000, Active Motif, Carlsbad, CA, USA #61017), mouse anti-HP1 (1:500, 9A9), mouse anti-αtubulin antibody (1:4000, Sigma, Merk Life Science S.r.l., Milan, Italy #T5168), rabbit anti-H3 pan-acetyl (1:1000, Millipore, Merk Life Science S.r.l., Milan, Italy #06-599), and rat anti- HSP70 clone 7Fb (1:5000, kindly provided by M. B. Evgen’ev). Proteins of interest were detected with horseradish peroxidase-conjugated anti-mouse, anti-rat and/or anti-rabbit, diluted 1:10,000 in TBST and visualized with the ECL Western blotting substrate (GE Healthcare, Chicago, IL, USA), according to the provided protocol. The chemiluminescence detection was performed on the ChemiDoc XRS+ System (Bio-Rad Laboratories S.r.l., Milan, Italy) and analyzed using ImageJ software version 1.53t. Results of independent biological replicates were compared and analyzed by the Unpaired *t*-test using GraphPad Prism version 6.00. A *p* value ≤ 0.05 was considered statistically significant.

### 2.6. Measurement of Eye Pigment

Heads of 60 male flies (2–3 days old, raised at 25 °C) from each experimental condition were homogenized in a final volume of 2 mL of methanol (acidified with 0.1% HCl). After centrifugation, the supernatant was removed and placed in cuvettes. Absorbance was measured at 480 nm using the Multiskan GO Spectrophotometer (Thermo Scientific’s, Waltham, MA, USA). Statistical comparisons were performed using the Paired *t*-test (GraphPad Prism version 6.00) with *p* ≤ 0.05 considered as statistically significant. The eyes of representative individuals were photographed using a Nikon camera D5000 mounted onto a stereoscopic microscope.

### 2.7. Mitotic Chromosome Preparations

Cytological preparations of mitotic chromosomes from *Drosophila* larval brain were obtained according to Pimpinelli et al. (2000) [65] and stained with DAPI (4,6-diamidino-2-phenilindole, 0.01 mg/mL) to visualize DNA. The slides were mounted in antifading medium (23.3 mg/mL of DABCO (1,4-Diazobicyclo-(2,2,2) octane) in 90% glycerol–10% PBS1X). All images were taken with an ellipse epifluorescence microscope (E1000 Nikon, Tokyo, Japan) equipped with a CCD camera (Coolsnap). Images were analyzed and further processed using Adobe Photoshop CS6. Statistical significance of chromosomal abnormalities was determined by the Chi-Square test using GraphPad Prism Software version 6.00. A *p* value ≤ 0.05 was considered statistically significant.

### 2.8. H2DCFDA Staining

Larval brains were dissected from third instar larvae in PBS1X. H2DCFDA was detected as previously described [66]. In brief, a PBS1X buffer containing 10 μM H2DCFDA (Thermo Fisher Scientific, Waltham, MA, USA) was added to larval brains, followed by incubation for 10 min in a dark chamber on an orbital shaker at room temperature. Excess H2DCFA was removed by performing three 5-min washes in PBS1X on an orbital shaker at room temperature. Subsequently, the samples were mounted in Vectashield. All images were taken with an ellipse epifluorescence microscope (E1000 Nikon, Tokyo, Japan) equipped with a CCD camera (Coolsnap). Images were analyzed and further processed using ImageJ/Fiji plugin to count spots (Spot Counter Plug-in version 0.14). Statistical significance was determined using the Unpaired *t*-test (GraphPad Prism Software version 6.00). A *p* value ≤ 0.05 was considered statistically significant.

### 2.9. Larval Crawling Assay

Larval crawling abilities were examined as reported previously [67,68]. Briefly, five third instar larvae for each group were transferred to a 15-cm Petri dish containing 2% agarose in PBS1X and left for 1 min to acclimate. Then, they were filmed for 1 min using a smartphone and their crawling behavior was tracked for 30 s with the help of a black graph paper as a reference measure. After pixel to mm conversion, the total distance travelled by larvae was calculated to determine the average speed of larvae in mm per second by using Imagej/WrmTrack plugin (v1.04). Travelled paths were obtained by enabling the ‘show paths’ box during wrMTrck analysis. The experiments were carried out in eight replicates and 40 larvae were analyzed for each experimental group. Statistical significance of crawling patterns was determined using the Unpaired *t*-test (for speed, total length travelled and distance) and Fisher’s exact test (for travel paths). A *p* value ≤ 0.05 was considered statistically significant.

### 2.10. Larval Light Preference Test

A larval light preference test was carried out as described by [69]. For this experiment, 2% agarose in PBS1X was plated in a Petri dish divided into four quadrants. The alternative quadrant was painted black. Before the experiment, 20 third instar larvae of each treatment group were placed in the dark for 6 h. After 6 h, larvae were transferred into the Petri dish and the lid of Petri dish was closed, thus having the same alternative black quadrant marking. The Petri dish was then placed under homogeneous light conditions (at 620 lux). For the next 5 min, the larvae were allowed to crawl between black and transparent quadrants. After 5 min, the number of larvae in each quadrant was counted by opening the Petri dish lid. The experiments were carried out in five replicates and 100 larvae were analyzed for each experimental group. Larval light preference index (LPI) was calculated as: number of larvae in the light half/(number of larvae in the dark half + number of larvae in the light half). LPIs were shown as mean ± SEM. Statistical significance was determined using Paired *t*-test (GraphPad Prism Software version 6.00). A *p* value ≤ 0.05 was considered statistically significant.

### 2.11. Climbing Assay

The climbing assay was performed as previously described [70]. Briefly, for each group, 10 flies were collected and allowed to recover for 2 h to avoid effects due to CO_2_ anesthesia. Then, they were flipped into a glass tube (9.5 cm × 2.5 cm), and the bottom was gently tapped. The number of flies that reached the 8 cm line after 10 s were counted. Ten trials were performed for each group and n ≥ 100 flies were assayed for each treatment. Experiments were performed during daylight to minimize potential effects of circadian oscillation. All average data are presented as mean ± SEM and compared with Unpaired *t*-tests. Statistical tests were performed using GraphPad Prism Software version 6.00. A *p* value ≤ 0.05 was considered statistically significant.

## 3. Results and Discussion

### 3.1. WiFi Electromagnetic Fields Induce Aberrant Expression of Transposable Elements

**Figure 1:**
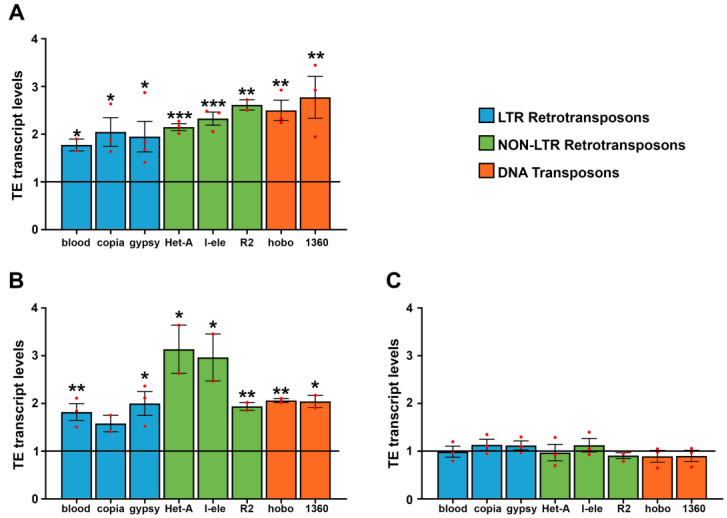
qRT-PCR analysis of transposable element expression in adult heads (**A**), ovaries (**B**) and testes (**C**) from control and WiFi-exposed 10 days-old flies. Transcript levels were normalized to *rp49* and displayed as fold change relative to the controls. Bar graph represents the mean ± SEM from at least two independent experiments (* *p* ≤ 0.05, ** *p* ≤ 0.01, *** *p* ≤ 0.001; Unpaired *t* tests). Red dots indicate individual data points. The black horizontal line indicates the fold change control value, set to 1.

To investigate the effects of WiFi on the activity of transposable elements, Oregon-R flies were exposed to 2.4 GHz radiofrequency throughout their development (from embryo through to adult stage). Total RNA from 10-day-old fly heads was purified, and the expression profiles of different families of transposable elements were analyzed by qRT-PCR. We initially profiled eight transposable elements, including two DNA transposons (*hobo* and *1360*), three retroviral-like Long Terminal Repeat (LTR) retrotransposons (*blood, copia* and *gypsy*), and three LINE-like non-LTR retrotransposons (*Het-A, R2* and *I-element*). The results of this analysis showed a significant upregulation for all transposable elements tested (Figure 1A).

To evaluate whether WiFi exposure derepresses transposable elements in germline tissues as well, we measured transcriptional activities of TEs in ovaries of 10-day-old flies exposed to WiFi radiation daily, starting from day two after eclosion. The results showed a weak but statistically significant upregulation for all transposable elements analyzed (Figure 1B), although LINE-like TEs *Het-A* and *I-element* were more differentially regulated in comparison to other TEs. We also examined transposon transcript levels in adult testes and failed to find any statistically significant differences between WiFi exposed and unexposed control flies (Figure 1C). Taken together, these results suggest that adult neuronal tissues and female germline are more susceptible than male germline to the release of transposon silencing upon WiFi exposure. This result is very interesting because it indicates that, following WiFi exposure, adult testes exert a regulation of TE silencing tighter than head and female germline tissues. Further studies will be needed to clarify the molecular and cellular mechanisms underlying the susceptibility of different tissues to electromagnetic fields.

We previously demonstrated that, in *Drosophila* germline, the inducible HSP70 chaperone is a key mediator of stress-induced transposable element activation. We found that, following prolonged heat shock, HSP70 chaperone partially displaces the HSP70-HSP90 piRNA complex to the lysosomes, thus resulting in a functional collapse of the piRNA pathway and in active TE mobilization [6,7]. 

**Figure 2:**
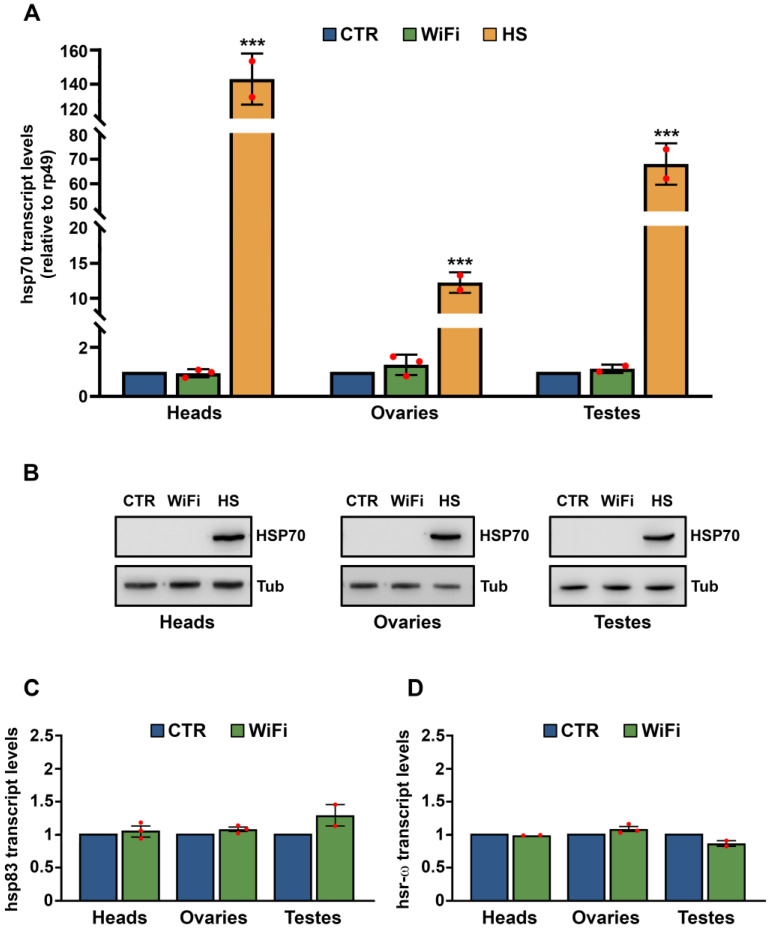
qRT-PCR (**A**) and western blot analysis (**B**) of *Hsp70* gene expression in adult heads, ovaries and testes from control, WiFi-exposed and heat shocked (HS) flies. (**A**) Transcript levels were normalized to rp49 and displayed as fold change relative to the controls. The bar graph represents the mean ± SEM from at least two independent experiments. Red dots indicate individual data points ( *** *p* ≤ 0.001; Unpaired *t* test). (**B**) HSP70 protein levels were normalized to α-Tubulin. qRT-PCR analysis of *Hsp83* (**C**) and *hsr-ω* (**D**) transcripts in adult heads, ovaries, and testes from control and WiFi-exposed flies. Transcript levels were normalized to *rp49* and displayed as fold change relative to the controls. Bar graph represents the mean ± SEM from at least two independent experiments. Red dots indicate individual data points.

Based on these previous studies that identify HSP70 protein as a positive regulator of stress-induced TE upregulation, we evaluated whether WiFi exposure could induce HSP70 expression next. We analyzed the expression profiles of *Hsp70* transcripts in heads, ovaries, and testes collected from control and WiFi exposed flies. As a positive control, we evaluated *Hsp70* gene expression following heat shock. As shown in Figure 2A, *Hsp70* transcripts were strongly upregulated in all heat shocked samples; on the contrary, no statistically significant differences in *Hsp70* mRNA levels were found between control and WiFi samples in all tissues analyzed.

The basal level of *Hsp70* transcript we detected by qRT-PCR in control and WiFi tissues was extremely low (as compared to *hsp70* transcript levels following heat stress) and does not encode a functional HSP70 protein (Figure 2B). We also evaluated the expression profiles of *Hsp83* (the *Drosophila* homolog of the mammalian *Hsp90*) and *hsr-ω*, two other important genes involved in thermal stress response in *Drosophila*. As shown in Figure 2C, and 2D, we did not find any significant difference between the transcriptional levels of control and WiFi flies among all tissues analyzed.

These results strongly suggest that electromagnetic field exposure can induce TE activation through a molecular mechanism independent of HSP70 induction. More importantly, they allow us to exclude any possible thermal effects of radiofrequency electromagnetic fields on transposable element deregulation.

### 3.2. A Heterochromatin Breakdown Contributes to the Transcriptional Activation of Transposable Elements

Several families of genetically active transposable elements, most of them belonging to the retrotransposon category, are stable structural components of constitutive heterochromatin in *Drosophila*. The highly condensed heterochromatin structure ensures transcriptional epigenetic silencing of repetitive sequences, such as satellite sequences and transposable elements [71].

To verify whether the aberrant upregulation of transposable elements in the heads of WiFi exposed flies could be associated with a perturbation in heterochromatin structure and function, we used a Position Effect Variegation (PEV) assay, a widely accepted genetic tool in understanding the dynamic of heterochromatin state in *Drosophila* [72]. Position effect variegation results when a euchromatic gene, juxtaposed with constitutive heterochromatin by chromosomal rearrangements, is transcriptionally silenced in cells but active in others, producing a diagnostic variegated, phenotype [73,74]. Since the variegating phenotype is caused by heterochromatin-induced gene silencing, all environmental factors that affect the structure and organization of pericentric heterochromatin could act as dose-dependent modifier of PEV-based gene silencing. 

**Figure 3:**
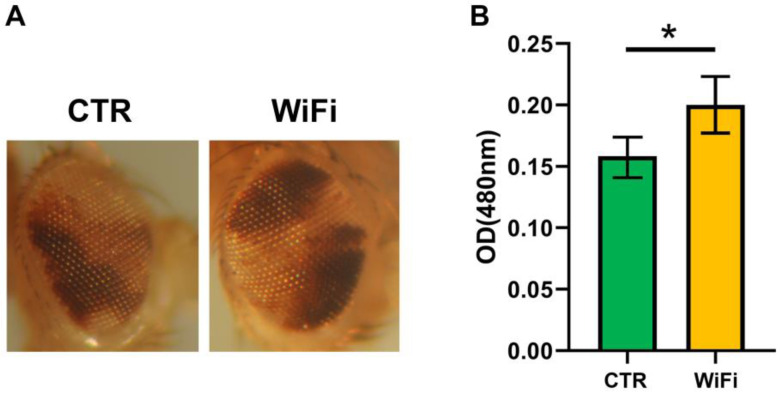
(**A**) Images of the BL2 PEV pattern in the adult fly eye taken from randomly selected individuals in each experimental group. (**B**) Quantitative assessment of pigment levels in the adult fly eyes from 2–3-days-old control and WiFi-exposed BL2 males. Statistical significance was determined using the Paired *t* test (* *p* ≤ 0.05).

To determine whether WiFi exposure affects heterochromatin structure, thus causing aberrant expression of TEs, we evaluated the PEV phenotype of the BL2 reporter line resulting from a translocation carrying two different variegating transgenes, an inducible *Hsp70-lacZ* and a mini-white reporter gene, into Y pericentromeric heterochromatin [75]. The expression level of mini-white variegating transgene was quantified by optical measurement of eye pigment extracts from groups of 60 exposed and control male heads (Figure 3). We were unable to analyze the expression of the inducible variegating transgene *Hsp70-lacZ* due to the inability to perform heat shock treatments inside the TEM chamber. The results of the PEV assay clearly demonstrated that WiFi exposure acts as a suppressor of position effect variegation, thus suggesting that WiFi induces TE upregulation through a widespread heterochromatin decondensation. We also tested the effects of WiFi EMF on the variegation of the *white (w)* gene associated with the *In(1)^wm4^* [73]. In the *In(1)^wm4^* rearrangement, the X chromosome contains an inversion that relocates the euchromatic white gene to the pericentric constitutive heterochromatin. Consistently with the PEV phenotype of the BL2 reporter line, WiFi exposure suppresses the PEV of *w^m4^* as well.

**Figure 4:**
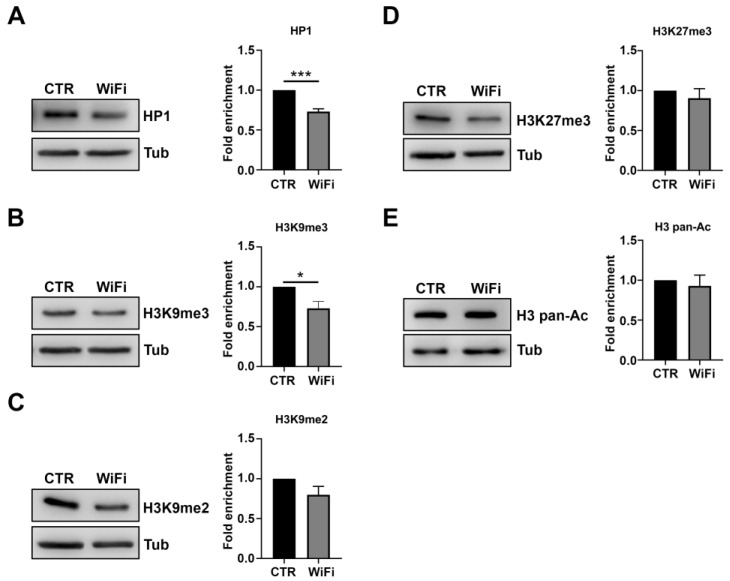
Western blot analysis of HP1 (**A**), H3K9me2/3 (**B**,**C**), H3K27me3 (**D**) and H3 pan-acetylated (**E**) in control and WiFi exposed flies. α-Tubulin protein was used as a loading control. The histograms on the right show results represented as mean values of at least two independent biological replicates (* *p* ≤ 0.05; *** *p* ≤ 0.001, Unpaired *t* test). Expression levels in control samples are set as one (black bar).

Trimethylation of histone H3 on lysine 9 (H3K9me2/3) and HP1 are representative epigenetic hallmarks of constitutive heterochromatin in *Drosophila* and other organisms [76,77,78]. Consistently with PEV results indicating a widespread heterochromatin relaxation following WiFi exposure, we found a significant decrease of HP1 and H3K9me3 levels in exposed adult heads as compared to unexposed controls (Figure 4A,B). Similarly, H3K9me2 protein levels tended to be reduced in the exposed flies but did not reach statistical significance (Figure 4C).

Notably, WiFi exposure specifically affects heterochromatin epigenetic marks without altering other epigenetic markers such as trimethylation of histone H3 at Lys-27 (H3K27me3), correlated with facultative heterochromatin or acetylation of histone H3 classically associated with transcriptionally active chromatin (Figure 4D,E). Taken together, these data strongly suggest that WiFi radiation could induce transposable element upregulation by affecting the structure and function of constitutive heterochromatin.

### 3.3. WiFi Radiation Induces Genome Instability in Larval Brains

A loss of heterochromatin integrity can generate genome stability defects by making chromatin more susceptible to DNA damage [79]. For instance, changes in heterochromatin components can alter the nuclear compaction of DNA sequences, thereby increasing susceptibility to DNA damage. Alterations in heterochromatic histone modifications can also directly affect DNA damage repair efficiency since many histone modifications have been implicated in promoting or inhibiting the recruitment of specific repair proteins [80]. In addition to heterochromatin destructuration, transposition events could be a further source of DNA strand breakage, which compromises genome integrity and stability [81,82,83,84]. Therefore, to verify whether WiFi radiation can impact genomic stability, we analyzed metaphase chromosomes obtained from brains of third instar larvae chronically exposed to WiFi radiation (from embryo to L3). 

**Figure 5:**
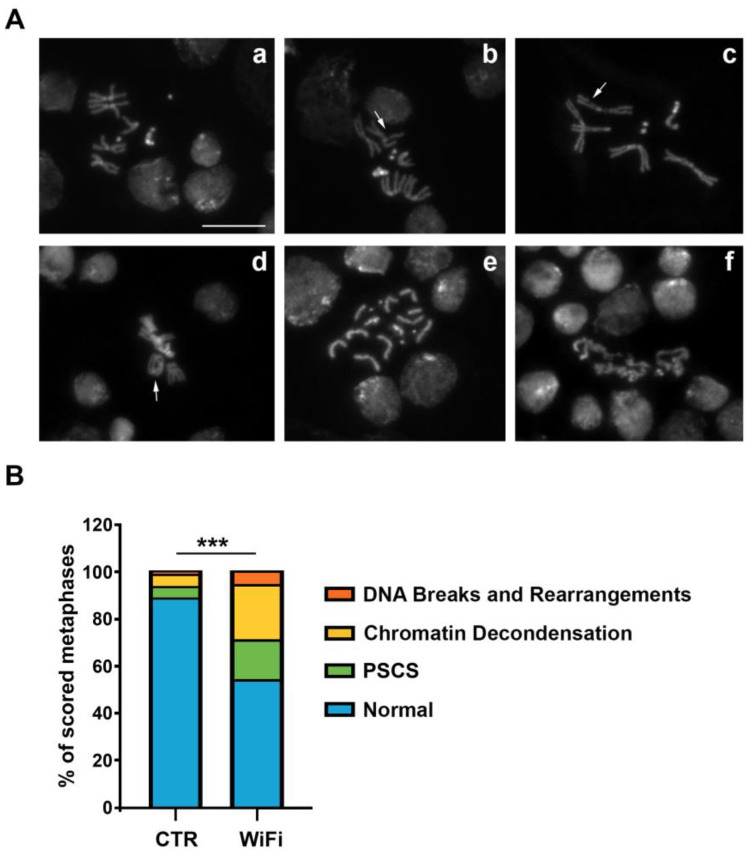
(**A**) DAPI-stained mitotic chromosomes obtained from control (**a**) and WiFi exposed (**b**–**f**) third-instar larval brains; (**a**) normal male metaphase; (**b**–**f**) examples of chromosome aberrations: DNA breaks (as indicated by arrows in **b**–**c**), DNA rearrangement (as indicated by arrow in **d**), PSCS (**e**) and chromatin decondensation (**f**). Scale bar indicates 10 μm. (**B**) Quantification of chromosomal abnormalities observed in control and WiFi-exposed larval brains (*** *p* ≤ 0.001, Chi-square test). PSCS, Precocious Separation of Sister Chromatid. At least 200 metaphases were scored in each of three independent experiments.

We found that WiFi exposure induces abnormal chromosome configurations such as breakages (Figure 5A(b,c)), chromosomal rearrangements (Figure 5A(d)), precocious sister chromatid separation (PSCS) (Figure 5A(e)), and a higher degree of both chromatin decondensation (Figure 5A(f)).

Importantly, the precocious sister chromatid separation could be related to the defects of pericentric heterochromatin structure and organization. In fact, previous genetic evidence from fission yeast, *Drosophila*, and mammalian cells indicated that correct heterochromatin structure is important for proper centromere function, as mutations that perturb heterochromatin at these regions also cause defects in cohesion of sister chromatid and chromosome mis-segregation [85,86,87,88,89]. For example, the degradation of *Drosophila* heterochromatin protein 1 (HP1), a structural component of silent heterochromatin, causes unbalanced chromosome segregation [86]. In fission yeast, Swi6, a homologue of HP1, is important for maintaining Scc1/Rad21 at the centromere until anaphase [90,91]. In humans, it has been reported that the dominant-negative form of HP1-β is involved in centromere cohesion [92] and that epigenetic displacement of both HP1-α and HP1-γ from centromeric heterochromatin causes premature sister chromatid separation [93,94]. Considering these results, we can assume that the centromere cohesion impairment we observed in exposed larval brains could result from epigenetic disruption of higher-order structures of constitutive heterochromatin.

**Figure 6:**
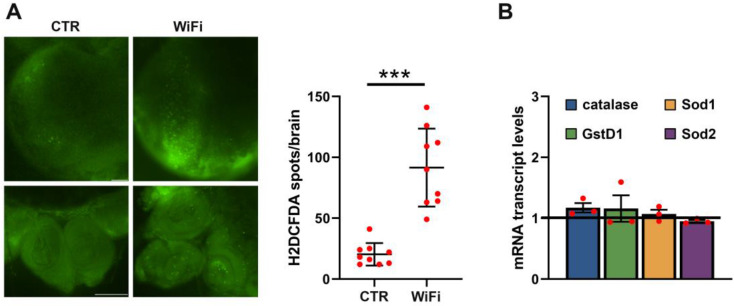
(**A**) Fluorescence microscope images of H2DCFDA in optic lobes (upper panels) and imaginal discs (lower panels) from control and WiFi exposed third instar larvae. The scale bar indicates 100 μm. The scatter plot indicates the quantitative analysis of H2DCFDA positive puncta. Data are the means ± SEM. The dots indicate individual data points across three independent biological replicates (*** *p* ≤ 0.01; Unpaired *t* test). (**B**) qRT-PCR analysis of ROS-related genes in control and WiFi-exposed larval brains. Transcript levels were normalized to rp49 and displayed as fold change relative to the unexposed controls. Bar graph represents the mean ± SEM from at least two independent experiments. Red dots indicate individual data points. The black horizontal line indicates the fold change control value, set to one.

We next considered mechanisms in which WiFi could induce heterochromatin loss and DNA damage. In *Drosophila*, oxidative stress promotes heterochromatin decondensation, genome instability, premature loss of cohesion, and chromosome segregation defects [95,96,97]. Therefore, to verify if WiFi exposure could induce oxidative stress by triggering heterochromatin loss and DNA damage, we assessed in vivo ROS levels in *Drosophila* larval brains using the ROS-reactive dye 2′,7′dichlorodihydrofluorescein diacetate (H2DCFDA) [66] (Figure 6).

The results of this analysis showed that long term exposure to WiFi radiation (from embryo to third instar larval stage) causes a moderate but significant increase in endogenous ROS levels (Figure 6A). We also examined the expression profiles of key genes involved in oxidative stress response by qRT-PCR, but did not find any statistically significant difference between control and WiFi exposed larval brains (Figure 6B), suggesting that, in this case, the protection from oxidative stress could be achieved by an increased activity of these antioxidant enzymes rather than by their transcriptional upregulation. Collectively, these findings lead us to speculate that WiFi exposure induces oxidative stress and heterochromatin loss. Together, this could trigger TEs activation and genetic instability.

### 3.4. WiFi Exposure Impairs Locomotor Behaviour in Larvae and Adult Flies

It is well known that DNA damage and genomic instability causes apoptosis, which, in turn, represents the main cause of neurodegeneration [98]. A typical hallmark of neurodegeneration is progressive and severe locomotor impairment, as locomotor behavior relies on complex neural circuits involving multiple interacting networks of both motor and sensory neurons. For this reason, any neural defect is reflected as defective locomotor behavior.

To assess the neurodegeneration levels following WiFi exposure, we characterize and quantify the locomotor behavior of third instar larvae and adult flies by using crawling and climbing assays, respectively. The larval crawling assay is an effective and reliable assay to quantify larval locomotion activity that is controlled directly by the motor neurons in the larval brain [67,99].

**Figure 7:**
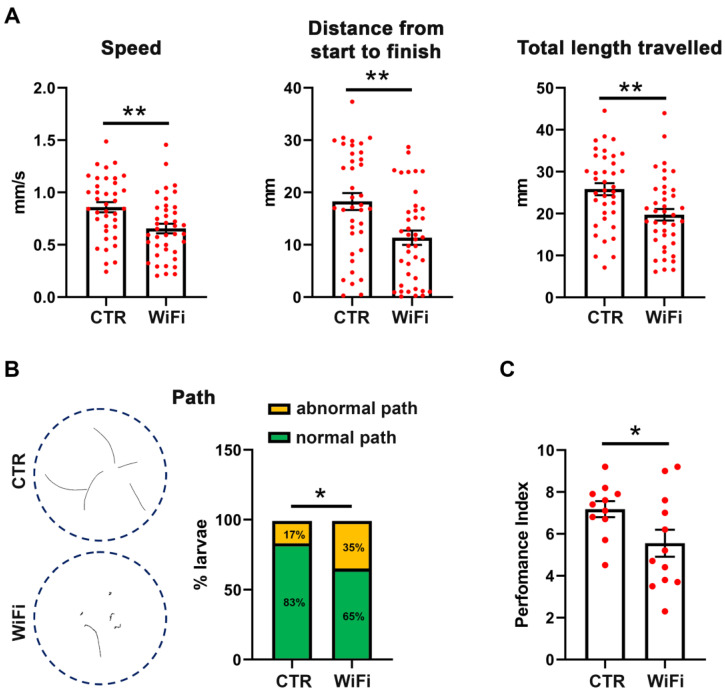
(**A**) Larval crawling patterns of control and WiFi-exposed third instar larvae. Quantitation of the speed was in millimeters per second, distance from start to finish, and total length travelled (speed × time) were in millimeters. Red dots indicate individual data points from eight independent biological replicates. Data were analyzed using the Unpaired *t* test (** *p* ≤ 0.01). (**B**) Schematic illustration and quantification of larval trailing paths following WiFi exposure. Statistical significance was determined by Fisher’s exact test (* *p* ≤ 0.05). (**C**) Climbing assay on 10-days old unexposed control and WiFi exposed flies. Red dots indicate individual data points from two independent biological replicates. Statistical significance was determined using the Unpaired *t* test (* *p* ≤ 0.05).

As shown in Figure 7A, the crawling speed of WiFi exposed larvae were significantly compromised when compared with control larvae (0.86 ± 0.05 mm/s for the control vs 0.65 ± 0.05 mm/s for WiFi). We also analyzed the distance from start to finish and the total length travelled by each larva and found that exposed larvae ended up closer to their starting points and travelled shorter overall distances than controls (distance from start to finish: 18.27 ± 1.62 mm for the control vs 11.36 ± 1.36 mm for WiFi; total length travelled: 25.84 ± 1.45 mm for the control vs 19.73 ± 1.39 mm for WiFi). To study the crawling behavior in more detail, we further evaluated the larvae trailing paths. As reported in Figure 7B, control larvae travelled relatively straight paths, whilst exposed larvae exhibited shorter overall distances and jagged or circular paths. The rate of abnormal travel paths was 35% in WiFi exposed larvae and 17% in unexposed control larvae.

Consistently with the crawling assay, climbing behavior of adult flies monitored after 10 days of WiFi exposure was significantly impaired (Figure 7B). The performance index was 7.16 ± 0.38 for control and 5.55 ± 0.65 for WiFi exposed flies.

**Figure 8:**
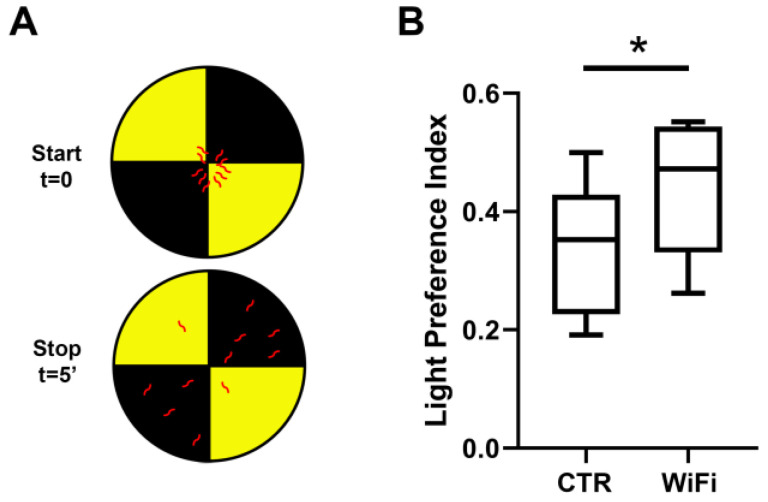
(**A**) Light preference test, example of a dish right after setting larvae on the test plate (Start) and after 5 min (End). (**B**) Percentage of larvae that preferred light were counted for each trial and all trials were averaged. The experiments were carried out in five independent biological replicates and 100 larvae were analyzed for each experimental group. Statistical significance was determined using the Paired *t* test (* *p* ≤ 0.05).

To evaluate any early defect in the light-sensing neurons, we next carried out a light preference test based on the stereotyped photophobic behavior of the larvae when exposed to light [69] (Figure 8). The control larvae showed a light preference index of 0.33 ± 0.05, while WiFi exposed larvae showed a light preference index of 0.44 ± 0.05, suggesting that WiFi RF-EMFs negatively regulates the larval light avoidance photo behavior. This likely interferes with the circadian photoreceptor cryptochrome (CRY), a *Drosophila* magnetosensor implicated in the EMF responses of the flies [100,101,102].

### 3.5. WiFi Radiation Promotes Metastatic Behaviour of Non-Invasive Eye Disc Clones Expressing the Activated Oncogene Ras^V12^

It is well known that chromatin alteration and uncontrolled TE mobilization can have a strongly mutagenic effect on genomes, contributing substantially to the onset, development, and metastatic progression of tumors [103]. Moreover, numerous but also controversial experimental and epidemiological evidence suggests that prolonged exposure to radio frequencies significantly increases the risk of intracranial tumors, in particular, gliomas, meningiomas, and acoustic neuromas [104,105]. Based on these studies, the Agency for Research on Cancer (IARC) has classified radiofrequency electromagnetic fields as possibly carcinogenic to humans (Group 2B). In recent years, many transgenic models of cancer have been developed in *Drosophila* [106,107,108]. In flies, there are many genes related to cancer and for which human functional homologues have been identified, such as oncogenes, tumor suppressors and genes whose mutation causes neoplastic growth [106,108]. It has also been shown that many signaling pathways are conserved from flies to humans, giving *Drosophila* functional relevance in the study of human cancer biology [106,109,110].

To evaluate whether chronic WiFi exposure can promote the progression of epithelial tumors and increase their metastatic potential, we used a sophisticated genetic approach of somatic recombination (MARCM, Mosaic Analysis with a Repressible Cell Marker) [60] that allowed us to generate tumor somatic clones overexpressing the Ras oncogene (*Ras^V12^*) in the eye-antennal discs. We analyzed their invasive potential by exploiting the simultaneous expression of GFP (Green Fluorescent Protein). We crossed females *ey-FLP1; act > y^+^ > Gal4, UAS-GFP; FRT82B, tub-Gal80* (MARCM 82B tester line) with males carrying the *UAS-Ras^V12^* transgene, distal to FRT site (*FRT82B, UAS-Ras^V12^*). The tumor somatic clones overexpressing *Ras^V12^* were produced through a specific somatic recombination of the FRT sequences by the enzyme Flippase (FLP) which, expressed under the control of the *eyeless* promoter, allowed us the overexpression of *Ras^V12^* driven by *actin-Gal4*, in only the recombinant cells that received the *UAS-Ras^V12^* construct, losing instead the transgene encoding Gal80 [60]. 

**Figure 9:**
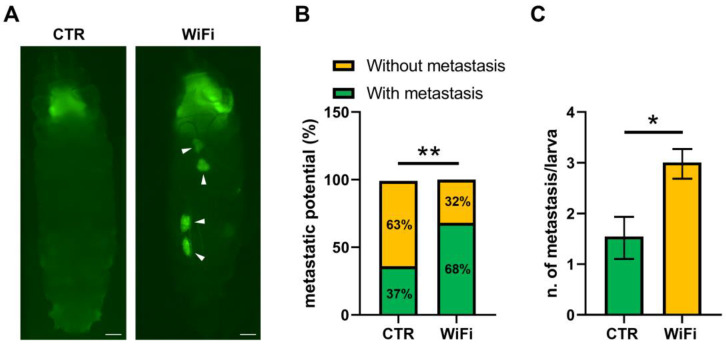
(**A**) WiFi exposure promotes overgrowth and metastasis of GFP-positive *Ras^V12^* cells. Arrowheads indicate migrating tumors. Scale bars indicate 200 μm. (**B**,**C**) Analysis of metastatic behavior in control and WiFi exposed larvae. About 100 larvae were analyzed from four independent biological replicates. Statistical significance was determined using Fisher’s exact test (**B**) and the Unpaired *t* test (**C**) (* *p* ≤ 0.05; ** *p* < 0.01).

As reported in Figure 9A, in control larvae, clones of cells expressing *Ras^V12^* in the developing eye exhibited benign overgrowth and rarely invaded into other tissues. Conversely, WiFi exposed larvae developed tumor overgrowth and metastasis of GFP-positive *Ras^V12^* cells (Figure 9A).

The rate of metastasis was 68% for WiFi exposed larvae and 37% for controls (Figure 9B); moreover, the mean number of metastases was 3 ± 0.26 in WiFi exposed larvae and 1.54 ± 0.39 in control larvae (Figure 9C). Collectively, these findings strongly suggest that WiFi RF-EMF synergized with oncogenic *Ras^V12^* in promoting tumor progression and invasion.

## 4. Conclusions

Over the last years, there has been mounting concerns about possible adverse health effects resulting from exposure to radiofrequency electromagnetic fields, such as those emitted by wireless communication devices. Currently, the studies conducted on exposure to high-frequency electromagnetic fields are numerous, but still not completely exhaustive and often discordant and contradictory. In fact, while some studies tend to underestimate the harmful effects of exposure to electromagnetic fields generated by WiFi transmissions, others instead clearly define the health risks associated with exposure to radio frequencies. Therefore, deepening the biological effects of electromagnetic fields arouses great interest not only in the scientific field but also in public opinion. Our work fits in this confused and evolving scientific context and establishes a new framework for discussing the biological effects of WiFi related radiofrequency electromagnetic fields on genomic stability, neurodegeneration, and tumorigenesis.

## Data Availability

Not applicable.
